# Changes in Smoking Behavior Since the Declaration of the COVID-19 State of Emergency in Japan: A Cross-sectional Study From the Osaka Health App

**DOI:** 10.2188/jea.JE20200533

**Published:** 2021-06-05

**Authors:** Shihoko Koyama, Takahiro Tabuchi, Sumiyo Okawa, Takayoshi Kadobayashi, Hisaya Shirai, Takeshi Nakatani, Isao Miyashiro

**Affiliations:** 1 Cancer Control Center, Osaka International Cancer Institute, Osaka, Japan; 2 Health Promotion Division, Department of Public Health and Medical Affairs, Osaka Prefectural Government, Osaka, Japan

**Keywords:** tobacco, cigarette, COVID-19, declaring the state of emergency

## Abstract

**Background:**

In April 2020, the Japanese government declared a state of emergency due to the COVID-19 pandemic, and infection control measures, including requests to work from home and stay-at-home restrictions, were introduced. This study examined changes in smoking behavior during the COVID-19 state of emergency.

**Methods:**

An online cross-sectional survey was conducted in Osaka, Japan. To assess differences in smoking behavior among 5,120 current smokers before and after the declaration of a state of emergency, prevalence ratios (PRs) for two outcomes, increased smoking and quitting smoking, were calculated using multivariable Poisson regression, adjusting for potential covariates.

**Results:**

We found 32.1% increased the number of cigarettes smoked and 11.9% quit smoking. After adjustment for all variables, we found risk factors for COVID-19 (men and older age group) had both significantly higher PR for quitting smoking (men: PR 1.38; 95% confidence interval [CI], 1.17–1.62) and participants aged ≥65 years: PR 2.45; 95% CI, 1.92–3.12) and significantly lower PR of increased smoking (men: PR 0.85; 95% CI, 0.78–0.93 and participants ≥65 years: PR 0.38; 95% CI, 0.29–0.49). Additionally, respondents working from home or living alone had significantly higher PR for increased smoking (working from home: PR 1.29; 95% CI, 1.17–1.41 and living alone: PR 1.23; 95% CI, 1.10–1.38) and respondents who changed from cigarettes to heated tobacco products (HTPs) had significantly lower PR for quitting smoking (PR 0.150; 95% CI, 0.039–0.582).

**Conclusions:**

We suggest people who have high-risk factors for COVID-19 might change their smoking behavior for the better, while people who work from home or live alone might change their smoking behavior for the worse, during the COVID-19 state of emergency. Additionally, changing from smoking cigarettes to using HTPs makes smokers less likely to quit.

## INTRODUCTION

The 2019 coronavirus disease (COVID-19) outbreak was first reported in Wuhan City, Hubei Province, China in late December 2019. Since then, the causative virus, severe acute respiratory syndrome coronavirus 2 (SARS-CoV-2) has spread rapidly worldwide; as of July 19, 2020, a total of 14,043,176 confirmed cases and 597,583 deaths had been reported globally.^1^ As of July 19, 2020, a total of 24,642 confirmed cases and 985 deaths had been reported in Japan^2^; of these, 2,420 cases and 86 deaths were in Osaka.^3^ Unfortunately, the COVID-19 pandemic is continuing to spread, and there is an urgent need for measures to limit the harmful effects of the virus.

Each national government has introduced infection control measures for COVID-19, such as school and workplace closures, stay-at-home restrictions, income support, and debt relief.^4^ These measures influenced not only “COVID19-infected people” but also the entire general population, including non-infected people. Smoking cessation and working-from-home were among the suggested measures which form the “new normal lifestyle”.^5^^,^^6^ Investigation of the health effects of the “new normal lifestyle” is warranted in each country.

In Japan, after an increase in the number of COVID-19 cases, the government declared a state of emergency in seven central prefectures, including Tokyo, Osaka, Kanagawa, Chiba, Saitama, Fukuoka, and Hyogo, on April 7, 2020.^7^^,^^8^ This was expanded to all the other prefectures on April 16, 2020.^9^^,^^10^ The Japanese state of emergency allows prefectural governors to ask residents to stay home but there is no penalty for non-compliance with this request. It can also request closure of schools, some child and senior care or community centers, and stores and businesses that are considered non-essential.^11^ Unlike the European or Wuhan-style lockdowns, the Japanese government can neither force private companies to close and citizens to stay indoors nor impose penalties for non-compliance.^12^ The prefecture-level state of emergency continued for 4 to 7 weeks, depending on the infection level in each prefecture.^13^^,^^14^

**Figure 1.**
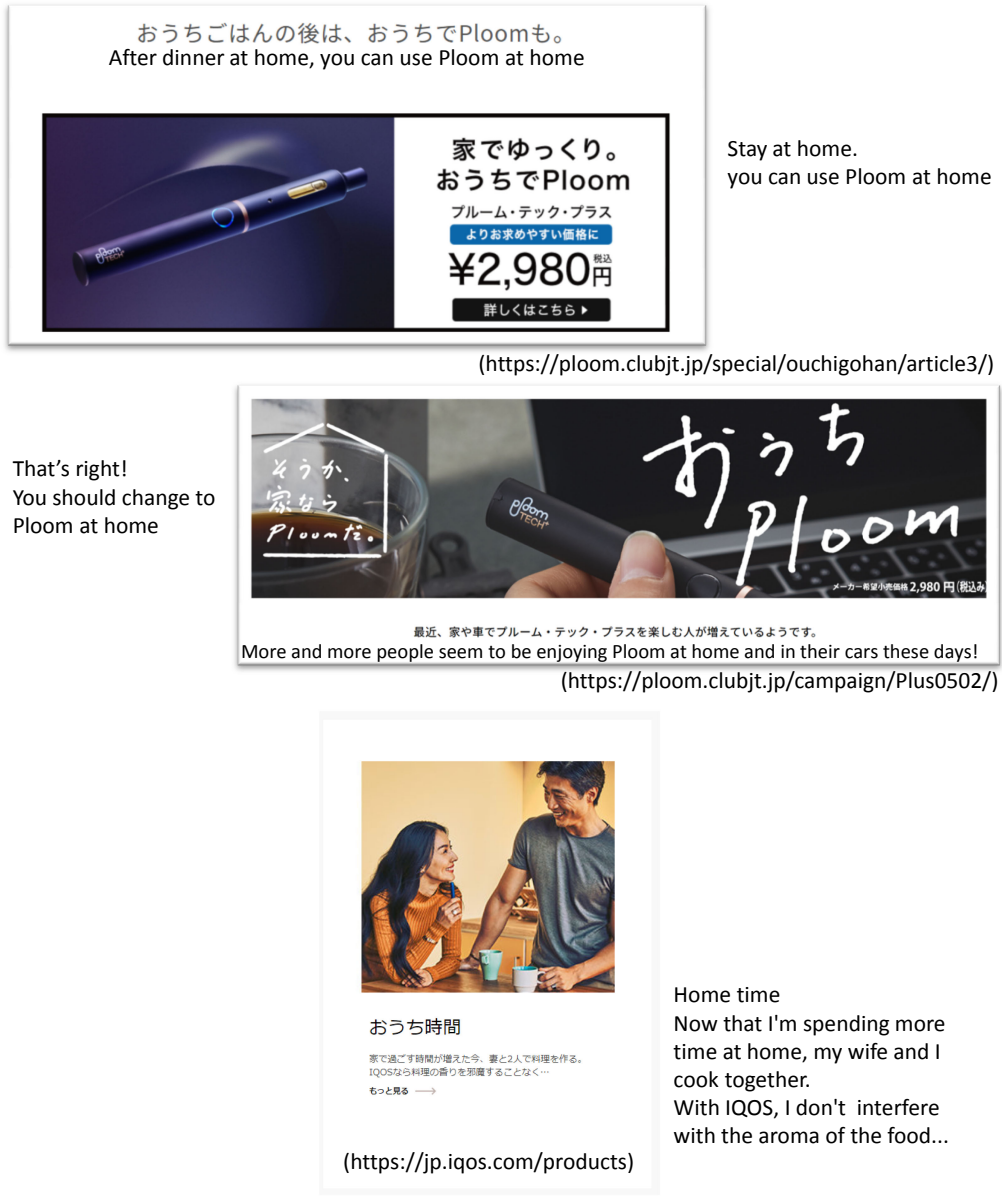
Tobacco industry promotion during COVID-19 Pandemic (URL)

In the first quarter of 2020, COVID-19 cases started rising in large numbers. During this period, three international Big Tobacco companies, Philip Morris International, Altria Group, and Japan Tobacco International, reported overall net revenue increases of 6.0%, 13.6%, and 2.8%, respectively.^15^ In Japan, during the state of emergency, tobacco companies ran advertising campaigns to encourage people who stay at home long-term to use heated tobacco products (HTPs) (Figure 1). For example, an IQOS starter kit, including five packs of tobacco sticks, was available for 2-weeks’ free rental in the Philip Morris campaign. Anyone could join this campaign online and would be sent the HTPs and the tobacco sticks without the need to leave their home.

Smoking might be one of the risk factors for COVID-19. According to several systematic reviews,^16^^–^^20^ compared to former and never smokers, current smokers were at higher risk of severe complications and a higher mortality rate from COVID-19. A previous study showed nearly 20% of respondents reported having seen claims that smoking can protect against COVID-19 in Hong Kong.^21^ Most smokers perceive themselves to be at higher risk of COVID-19 complications, and this risk appears to relate to intention to quit using tobacco and to attempting to quit.^22^ On the other hand, in some previous studies, more smokers reported increasing the number of cigarettes smoked rather than quitting or decreasing the amount they smoke due to COVID-19.^22^^–^^24^ In one study, 14.1% of smokers reported decreased smoking and 18.9% reported increased smoking.^24^

So far, there has not been a population-level observational study which examined the effect of the COVID-19 pandemic on smoking behavior in a large number of participants (over 1,000) and also considered a change from conventional cigarettes to HTPs. Few studies have examined the determinants of either increasing or quitting smoking due to COVID-19. During the COVID-19 lockdown, among patients with chronic coronary syndromes, the increase in number of cigarettes smoked was significantly greater in urban patients.^25^ Living alone more than doubled the odds of smoking more cigarettes during lockdown compared to living with others.^26^ Participants who had diabetes had a significantly higher odds ratio of wanting to quit smoking since the beginning of the pandemic.^27^ On the other hand, previous studies have reported that smoking behavior changes were unrelated to age,^28^^–^^30^ sex,^28^^–^^30^ population density,^29^ and living alone.^28^ While increased smoking is detrimental to health, quitting can improve health outcomes. Therefore, the objective of this study was to explore the effect of the COVID-19 state of emergency on smoking behavior change using data from the Osaka health application (app) survey. We examined factors for both increased smoking and quitting smoking.

## METHODS

### Study population

An online cross-sectional survey of people living in Osaka, Japan was conducted using the Osaka Prefecture health app^31^ between May 27 and June 14, 2020. This app is known as “Asmile” and is available to everyone living in Osaka Prefecture over the age of 18 who use a smartphone or tablet, who can download from Apple store or Google play (Language: Japanese only). “Asmile” was popularized by TV commercials^32^ during December 7–13, 2019, and websites^31^ from May 19, 2019 to the present. By downloading the app, people can record health activities, such as walking and joining in health events; however, participants cannot record smoking behavior under normal circumstances. Participating in these activities also allows the individual to accumulate points and, when sufficient points have been collected, participate in a lottery for prizes such as electronic money, food, and beverages. All the participants in this online-survey collected Asmile points to participate in the lottery.

Osaka Prefecture is located in the central part of Kansai, and has the third-highest population (8.82 million people) in Japan. In Osaka, the state of emergency was applied from April 7, 2020 to May 21, 2020. Initially, the Osaka Prefecture governor requested people to stop going out or attending open events (with no penalty for non-compliance). Daily necessities, such as medical visits, food shopping, and commuting to work, were allowed, although working from home (teleworking) was strongly encouraged.^33^ From April 14, the use of exercise facilities (eg, gymnasiums, swimming pools, bowling alleys, and sports clubs), recreation facilities (eg, nightclubs, bars, internet cafes, and karaoke boxes), education facilities (eg, schools), and theatres (eg, movie theatre) was also restricted. Restaurants, including pubs, were only allowed to open between 5:00 a.m. and 8:00 p.m., and to serve alcoholic beverages until 7:00 p.m.^34^

The online survey was designed to collect demographic information and details of smoking behavior change that occurred during the state of emergency. Approximately 195,000 people had installed the Asmile health app when the online survey started (May 27, 2020) and all users were invited to participate in the survey. The characteristics of the Asmile users were as follows: most users were in the 40–60 year age group, there were 1.5 times as many women users as men users, and the percentage of users in each municipality was 1.035–3.702%.^31^ When participants installed the health app, they provided the following web-based informed consent^35^: “Osaka Prefecture can use your basic registration information, personal health information, terminal information, and other information. Additionally, Osaka Prefecture can use participant’s data for the purpose of administrative measures for Osaka Prefecture or participating municipalities. Users who downloaded the app agree their data can be used for this purpose or disclosed as statistical information in a non-personally identifiable form.” Their age consistency was verified using some form of identification (eg, driver’s license and health insurance card). However, 120 participants did not provide such identification, so anyone with missing for age was excluded from the analysis. There were no missing responses in other variables.

### Outcome variables: increased smoking and quit smoking

We used changes in the number of cigarettes smoked, such as an increase, reduction, or quitting, as the main outcome variable for smoking behavior change. To assess these changes during the state of emergency, participants were initially asked: “What changes in health activities have you made following the declaration of the state of emergency to prevent the spread of the COVID-19?” We then asked specifically: “What about smoking behavior change?”. The possible responses were “increased”, no change”, “decreased”, and “quit”. The answer “increased” was defined as a dichotomized outcome of increased smoking (1 = increase, 0 = no change, decrease, quit). Similarly, the answer “quit” was defined as a dichotomized outcome of quit smoking (1 = quit, 0 = no change, decrease, increase).

### Explanatory variables

Covariates included the following variables: tobacco type change, sex (woman or man), age (<35, 35–44, 45–54, 55–64, and ≥65 years), number of people living together (1 (living alone), 2, 3, or 4 or more), change of income (increased, no change, decreased ≤24%, decreased 25–49%, decreased 50–99%, decreased 100%, or prefer not to answer), change of stress (no change, increased, or decreased), working from home (no or yes), employment type (full-time employee, part-time employee or other, not working), and population density of residential area (highest, high, low, or lowest). The tobacco type changes were assessed using the question: “What changes in health activities have you made following the declaration of a state of emergency to prevent the spread of the COVID-19? What about smoking behavior change?” The possible responses were “changed from cigarettes to heated tobacco products (HTPs)” and “changed from HTPs to cigarettes”. Respondents who did not choose either of these answers were defined as “no tobacco type change”. Working from home was assessed using the question: “What changes in health activities have you made following the declaration of a state of emergency to prevent the spread of the COVID-19? Have you changed to working from home?” The possible responses were “yes” and “no”. As an index of urban or local geography, population density was divided into quadrants using national census data^36^: highest, >11,900; high, 10,000–11,900; low, 5,000–9,999; or lowest, <4,999 residents per square kilometre.

### Statistical analysis

First, to observe simple relationships between smoking behavior changes and participant characteristics, cross-tabulation and chi-square or fisher’s exact test were performed. Second, we examined the associations between smoking behavior change and potential covariates using univariable and multivariable Poisson regression models. We chose Poisson regression using robust variance estimations because the prevalence of increased or quit smoking was more than 10% in our data.^37^ We calculated the prevalence ratios (PRs) and 95% confidence intervals (CIs) for smoking status based on the key variables of smoking behavior change.

All analyses were conducted using Stata version 15.1 (Stata Corp, College Station, TX, USA).

### Ethical considerations

The study was approved by the Institutional Review Board of the Osaka International Cancer Institute (approval number: 20102) before initiating the study. The data were anonymized by Osaka Prefecture before use in this study.

## RESULTS

**Figure 2.**
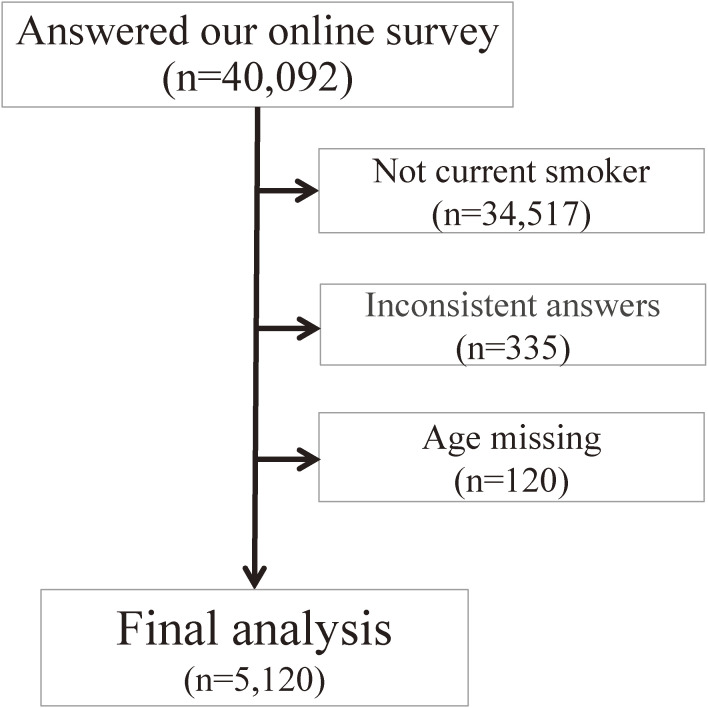
Data from 5,120 participants included in the analysis

Of the 40,092 total respondents in the online survey, 34,517 who were not current smokers, 335 who reported inconsistent answers (eg, chose both “no change” and “increased”) and 120 who had missing age were excluded from the analysis. Thus, 5,120 (2,505 women and 2,615 men) eligible participants remained in the analysis (Figure 2).

In terms of changes in smoking behavior during the COVID-19 state of emergency, we found that 32.1% of current smokers had increased the number of cigarettes smoked per day, while 11.9% had quit. Table 1 shows the characteristics of study participants according to smoking behavior change. Of current smokers, 2.2% changed from smoking cigarettes to HTPs, and 0.6% of current smoker changed from HTPs to cigarettes. Of smokers who changed from cigarettes to HTPs, 1.8% quit smoking. Of under-35-year-old smokers, 43.2% increased the number of cigarettes smoked, while 12.3% quit smoking. Of over-65-year-old smokers, 10.6% increased the number of cigarettes smoked, and 29.9% quit.

**Table 1.** Current smoker characteristics according to smoking behavior change (*n* = 5,120)

	Characteristics		Smoking behavioral change							

			Increased		No change		Decreased		Quit	
	*n*	column %	*n*	row %	*n*	row %	*n*	row %	*n*	row %
Total	5,120	100	1,645	32.1	2,292	44.8	576	11.3	607	11.9
Tobacco type change (Cigarettes to HTPs)									^*^	
No	5,007	97.8	1,607	32.1	2,248	44.9	547	10.9	605	12.1
Yes	113	2.2	38	33.6	44	38.9	29	25.7	2	1.8
Tobacco type change (HTPs to cigarettes)										
No	5,090	99.4	1,636	32.1	2,277	44.7	573	11.3	604	11.9
Yes	30	0.6	9	30.0	15	50.0	3	10.0	3	10.0
Sex			^*^						^*^	
Women	2,505	48.9	917	36.6	1,092	43.6	257	10.3	239	9.5
Men	2,615	51.1	728	27.8	1,200	45.9	319	12.2	368	14.1
Age, years			^*^						^*^	
<35	366	7.1	158	43.2	126	34.4	37	10.1	45	12.3
35–44	1,005	19.6	419	41.7	419	41.7	79	7.9	88	8.8
45–54	1,921	37.5	652	33.9	908	47.3	197	10.3	164	8.5
55–64	1,280	25.0	358	28.0	604	47.2	172	13.4	146	11.4
≥65	548	10.7	58	10.6	235	42.9	91	16.6	164	29.9
Number of people living together			^*^						^*^	
1 (living alone)	1,028	20.1	396	38.5	416	40.5	105	10.2	111	10.8
2	1,543	30.1	448	29.0	701	45.4	165	10.7	229	14.8
3	1,232	24.1	376	30.5	563	45.7	153	12.4	140	11.4
4 or more	1,317	25.7	425	32.3	612	46.5	153	11.6	127	9.6
Change of income			^*^							
Increased	68	1.3	19	27.9	31	45.6	10	14.7	8	11.8
no change	2,880	56.3	807	28.0	1,383	48.0	327	11.4	363	12.6
decreased ≤24%	886	17.3	327	36.9	375	42.3	98	11.1	86	9.7
decreased 25–49%	346	6.8	137	39.6	138	39.9	38	11.0	33	9.5
decreased 50–99%	403	7.9	153	38.0	156	38.7	49	12.2	45	11.2
decreased 100%	311	6.1	129	41.5	104	33.4	37	11.9	41	13.2
Did not want to answer	226	4.4	73	32.3	105	46.5	17	7.5	31	13.7
Change of Stress			^*^							
no change	1,838	35.9	415	22.6	973	52.9	210	11.4	240	13.1
increased	2,815	55.0	1,051	37.3	1,166	41.4	292	10.4	306	10.9
decreased	467	9.1	179	38.3	153	32.8	74	15.8	61	13.1
Work from Home			^*^						^*^	
No	3,920	76.6	1,181	30.1	1,837	46.9	410	10.5	492	12.6
Yes	1,200	23.4	464	38.7	455	37.9	166	13.8	115	9.6
Employment type			^*^						^*^	
full-time employee	2,470	48.2	842	34.1	1,120	45.3	282	11.4	226	9.1
part-time employee or other	1,935	37.8	635	32.8	873	45.1	205	10.6	222	11.5
not working	715	14.0	168	23.5	299	41.8	89	12.4	159	22.2
Population density of residential area			^*^						^*^	
Highest	1,946	38.0	680	34.9	840	43.2	221	11.4	205	10.5
High	605	11.8	191	31.6	289	47.8	61	10.1	64	10.6
Low	1,274	24.9	383	30.1	587	46.1	146	11.5	158	12.4
Lowest	1,295	25.3	391	30.2	576	44.5	148	11.4	180	13.9

HTPs, heated tobacco products.

^*^P < 0.05 for chi-square test or Fisher’s exact test.

After adjustment for covariates, significant associations with increased smoking were observed in men (PR 0.85; 95% CI, 0.78–0.93), younger age group (under 35 years old: PR 1.22; 95% CI, 1.06–1.39, 35–44 years old: PR 1.20; 95% CI, 1.09–1.32), older age group (55–64 years old: PR 0.84; 95% CI, 0.75–0.93, 65 years old or more: PR 0.38; 95% CI, 0.29–0.49), living alone (PR 1.23; 95% CI, 1.10–1.38), loss of income due to COVID-19 (decreased ≤24%: PR 1.20; 95% CI, 1.08–1.33, decreased 25–49%: PR 1.33; 95% CI, 1.15–1.54, decreased 50–99%: PR 1.37; 95% CI, 1.19–1.59, decreased 100%: PR 1.44; 95% CI, 1.23–1.68), change of stress (increased: PR 1.49; 95% CI, 1.35–1.64, decreased: PR 1.32; 95% CI, 1.14–1.53) and working from home (PR 1.29; 95% CI, 1.17–1.41) compared with their counterpart categories (Table 2). Tobacco type change, employment type, and population density of residential area were not significantly associated with increased smoking.

**Table 2.** Association between increased smoking and characteristics of current smokers using Poisson regression analysis (*n* = 5,120)

	univariable				multivariable			
	PR	95% CI		*P*-value	PR	95% CI		*P*-value
Tobacco type change (Cigarettes to HTPs)								
No	ref				ref			
Yes	1.05	0.81	1.36	0.727	1.04	0.81	1.34	0.757
Tobacco type change (HTPs to cigarettes)								
No	ref				ref			
Yes	0.93	0.54	1.61	0.805	0.99	0.59	1.69	0.984
Sex								
Women	ref				ref			
Men	0.76	0.70	0.82	<0.001	0.85	0.78	0.93	<0.001
Age, years								
<35	1.27	1.11	1.45	<0.001	1.22	1.06	1.39	0.004
35–44	1.23	1.12	1.35	<0.001	1.20	1.09	1.32	<0.001
45–54	ref				ref			
55–64	0.82	0.74	0.92	<0.001	0.84	0.75	0.93	0.001
≥65	0.31	0.24	0.40	<0.001	0.38	0.29	0.49	<0.001
Number of people living together								
1 (living alone)	1.19	1.07	1.33	0.002	1.23	1.10	1.38	<0.001
2	0.90	0.81	1.005	0.061	1.07	0.96	1.20	0.219
3	0.95	0.84	1.06	0.342	1.01	0.90	1.13	0.877
4 or more	ref				ref			
Change of income								
increased	1.00	0.68	1.47	0.988	1.02	0.69	1.51	0.909
no change	ref				ref			
decreased ≤24%	1.32	1.19	1.46	<0.001	1.20	1.08	1.33	<0.001
decreased 25–49%	1.41	1.23	1.63	<0.001	1.33	1.15	1.54	<0.001
decreased 50–99%	1.35	1.18	1.56	<0.001	1.37	1.19	1.59	<0.001
decreased 100%	1.48	1.28	1.71	<0.001	1.44	1.23	1.68	<0.001
Did not want to answer	1.15	0.95	1.40	0.159	1.17	0.97	1.42	0.107
Change of stress								
no change	ref				ref			
increased	1.65	1.50	1.82	<0.001	1.49	1.35	1.64	<0.001
decreased	1.70	1.47	1.96	<0.001	1.32	1.14	1.53	<0.001
Work from Home								
No	ref				ref			
Yes	1.28	1.18	1.40	<0.001	1.29	1.17	1.41	<0.001
Employment type								
full-time employee	ref				ref			
part-time employee or other	0.96	0.89	1.05	0.375	0.95	0.86	1.05	0.353
not working	0.69	0.60	0.80	<0.001	0.99	0.85	1.15	0.855
Population density of residential area								
Highest	1.16	1.04	1.28	0.005	1.02	0.92	1.13	0.758
High	1.05	0.91	1.21	0.543	0.98	0.86	1.13	0.821
Low	1.00	0.89	1.12	0.943	0.95	0.85	1.07	0.424
Lowest	ref				ref			

CI, confidence interval; HTPs, heated tobacco products; PR, prevalence ratio; ref, reference

Significant associations with quit smoking were observed in respondents who changed from cigarettes to HTPs (PR 0.150; 95% CI, 0.039–0.582), men (PR 1.38; 95% CI, 1.17–1.62), younger age group (under 35 years old: PR 1.48; 95% CI, 1.08–2.02), older age group (55–64 years old: PR 1.24; 95% CI, 1.001–1.54, ≥65 years old: PR 2.45; 95% CI, 1.92–3.12), decreased stress (PR 1.42; 95% CI, 1.09–1.86), not working (PR 1.57; 95% CI, 1.23–2.00), and highest of population density of residential area (PR 0.83; 95% CI, 0.69–0.998) compared with their counterpart categories (Table 3). Number of people living together and working from home were not significantly associated with quit smoking.

**Table 3.** Association between quitting smoking and characteristics of current smokers using Poisson regression analysis (*n* = 5,120)

	univariable				multivariable			
	PR	95% CI		*P*-value	PR	95% CI		*P*-value
Tobacco type change (Cigarettes to HTPs)								
No	ref				ref			
Yes	0.146	0.037	0.580	0.006	0.150	0.039	0.582	0.006
Tobacco type change (HTPs to cigarettes)								
No	ref				ref			
Yes	0.84	0.29	2.47	0.755	0.83	0.28	2.44	0.733
Sex								
Women	ref				ref			
Men	1.47	1.27	1.72	<0.001	1.38	1.17	1.62	<0.001
Age, years								
<35	1.44	1.06	1.96	0.021	1.48	1.08	2.02	0.013
35–44	1.03	0.80	1.31	0.841	1.04	0.81	1.33	0.751
45–54	ref				ref			
55–64	1.34	1.08	1.65	0.007	1.24	1.001	1.54	0.049
≥65	3.51	2.89	4.26	<0.001	2.45	1.92	3.12	<0.001
Number of people living together								
1 (living alone)	1.12	0.88	1.43	0.358	1.02	0.80	1.29	0.884
2	1.54	1.26	1.89	<0.001	1.18	0.95	1.46	0.132
3	1.18	0.94	1.48	0.157	1.06	0.85	1.33	0.609
4 or more	ref				ref			
Change of income								
increased	0.93	0.48	1.80	0.837	1.04	0.54	2.00	0.911
no change	ref				ref			
decreased ≤24%	0.77	0.62	0.96	0.021	0.92	0.73	1.15	0.444
decreased 25–49%	0.76	0.54	1.06	0.106	0.89	0.64	1.25	0.511
decreased 50–99%	0.89	0.66	1.19	0.416	1.00	0.74	1.35	0.985
decreased 100%	1.05	0.77	1.41	0.770	1.08	0.79	1.47	0.648
Did not want to answer	1.09	0.77	1.53	0.627	1.04	0.74	1.45	0.837
Change of stress								
no change	ref				ref			
increased	0.83	0.71	0.98	0.023	1.00	0.85	1.17	0.982
decreased	1.00	0.77	1.30	0.998	1.42	1.09	1.86	0.010
Work from Home								
No	ref				ref			
Yes	0.76	0.63	0.93	0.006	0.94	0.76	1.17	0.601
Employment type								
full-time employee	ref				ref			
part-time employee or other	1.25	1.05	1.49	0.011	1.22	0.997	1.50	0.054
not working	2.43	2.02	2.92	<0.001	1.57	1.23	2.00	<0.001
Population density of residential area								
Highest	0.76	0.63	0.91	0.004	0.83	0.69	0.998	0.047
High	0.76	0.58	0.995	0.046	0.80	0.62	1.05	0.104
Low	0.89	0.73	1.09	0.262	0.96	0.79	1.17	0.688
Lowest	ref				ref			

CI, confidence interval; HTPs, heated tobacco products; PR, prevalence ratio; ref, reference

## DISCUSSION

To the best of our knowledge, the present study is the first to report that more current smokers had increased the number of cigarettes smoked than had quit smoking during the COVID-19 state of emergency in Japan (32.1% increased smoking and 11.9% quit smoking). In a previous study of Japanese smokers in their 50s, about 7–8% of smokers quit smoking the following year.^38^ Compared with this figure, the percentage of quit smoking observed in the present study was slightly higher. The COVID-19 pandemic may have increased smoking cessation. In previous studies that have examined smoking behavior change during the COVID-19 pandemic, 45.2% of smokers increased their smoking in Poland, 40.9% in the United States, and 18.9% in the Netherlands.^22^^–^^24^ The COVID-19 state of emergency may have had a large negative influence on increased smoking, as in other disasters. After the Great East Japan Earthquake in 2011, 16.5% of smokers increased their smoking.^39^

Increased smoking is detrimental to people’s health while quitting smoking improves health outcomes. In the present study, we examined determinants of both increased smoking and quitting smoking. Factors significantly associated with increased smoking during the state of emergency were men, young age group (<45 years), older age group (≥55 years), living alone, decreaced income, changes in stress, and working from home. Factors associated with quitting smoking were tobacco type change (cigarettes to HTPs), men, youngest age group (<35 years), older age group (≥55 years), decreased stress, not working, and highest of population density of residential area.

Of smokers, 12% who smoked only cigarettes successfully quit, as opposed to 1.8% of smokers who made a change. Smokers who changed from cigarettes to HTPs were significantly less likely to quit smoking than those who did not change tobacco type (PR 0.15). This may be because some smokers were persuaded by tobacco industry promotions (Figure 1) to change to HTPs rather than quit. Although HTPs contain similar toxic substances to combustible cigarettes, many smokers believe the risks of smoking HTPs are lower because of these promotions.^40^^,^^41^

Smokers who were living alone or working from home were likely to increase their smoking. This is possibly because they were less likely to receive complaints from family members or co-workers about smoking or were not governed by workplace smoking restrictions. A previous study showed people who live alone were more likely to smoke than those living in three-generation households in Japan.^42^ An environment free from smoking rules or no fear of exposing others to second-hand smoke may have led to an increase in the number of cigarettes smoked by those working from home. The government strongly recommends “working from home” as a “new normal lifestyle” for infection control measures for COVID-19 but “quitting smoking” should also be strongly recommended as part of the “new normal lifestyle”. Men and older age group were associated with behavior changes for the better (ie, they were less likely to increase smoking and were likely to quit). This may be because concern about health is the most common reason for quitting smoking and these groups appeared to be at higher risk from COVID-19 infection.^43^^,^^44^ Before the state of emergency, a Chinese study reported that the rate of infection and mortality rate due to COVID-19 was higher in men than in women.^45^ Furthermore, the older age group has been reported to have a higher risk of severe COVID-19 complications and mortality than the younger age group.^46^^,^^47^ Compared with regular employees, people who did not work were more likely to quit smoking, which is consistent with previous studies.^48^

Smokers with decreased stress were more likely to quit smoking, and smokers with increased stress were likely to increase the number of cigarettes smoked per day. These results were consistent with a previous study which found that stress was associated with higher nicotine dependence.^49^ Surprisingly, smokers with decreased stress were also likely to increase the number of cigarettes smoked per day simultaneously. Potential reasons for this includes the increased opportunity for smoking created by working from home during the COVID-19 Pandemic.

The higher rate of lost income due to COVID-19 was associated with increased smoking. Loss of income may be a result of reduced working hours which would, in turn, lead to increased opportunities for smoking. Generally, a socioeconomic inequality in smoking behavior can be observed. People with lower incomes have a higher smoking prevalence and nicotine dependence (ie, increased smoking) than people with higher income.^50^^,^^51^ The results of the present study imply that loss of income due to COVID-19 may expand socioeconomic inequality in smoking.

### Limitations

The present study has several limitations. First, due to the cross-sectional design, causal interpretation of the association between smoking behavior change and the COVID-19 state of emergency was limited. Second, Osaka Prefecture is in the central part of Kansai, and has the third-highest population in Japan. However, the study only targeted one of the 47 prefectures in Japan and used an online self-reported survey; therefore, generalizability is limited. For example, people who are familiar with the smartphone app generally and with government campaigns to make people more active would be more likely to download the Asmile app and therefore the results might potentially be under or overestimated due to self-reporting and socially desirable bias. Third, misclassification of smoking behavior may occur, because it is a time dependent variable which might continue to change in complex ways during that time. However, in our study, the smoking behavior change was categorized as only four patterns of “increased” “no change” “decreased” and “quit”. Fourth, our survey lacks some important variables on health conditions, such as diabetes, cardiovascular disease, cancer, and nicotine therapy.

### Conclusions

During the state of emergency in Japan, 32.1% of smokers increased the number of cigarettes smoked per day, while 11.9% quit smoking. We suggest that people who have high-risk factors for COVID-19 might change their smoking behavior for the better, while people who working from home as part of the “New normal lifestyle” might change their smoking behavior for the worse. Additionally, smokers who changed from smoking cigarettes to using HTPs were less likely to quit smoking. Smoking is bad for health, regardless of whether people are at risk of COVID-19 or not; however, these results indicated a relevant need to recommend “quitting smoking” as a part of the “new normal lifestyle”.
